# Triangulation in biomedical research: navigating an ocean of uncertainty

**DOI:** 10.1007/s10654-025-01265-2

**Published:** 2025-07-01

**Authors:** Frank J. Wolters

**Affiliations:** Erasmus Medical Centre, Rotterdam, The Netherlands

**Keywords:** Triangulation, Epidemiology

## Introduction

A single study may provide some evidence, but generally far from proof. After all, no study is without risk of bias, and rarely does a single study answer on all the potentially relevant heterogeneity of effect within the wider population. How to enhance the robustness of scientific conclusions in light of this clear and present danger? In their recent article in the journal, Gutierrez et al. advocate a broader uptake of triangulation [1].

## From sea-faring to sociology

The concept of triangulation was introduced in sociology in the 1950s [2, 3]. Borrowing from navigation and cartography, the metaphor served to encourage researchers to approach their questions from multiple angles. In doing so, they could circumvent biases or methodological limitations of any single approach, in order to pinpoint the most likely answer to their question. The concept of triangulation has since been adopted in various other fields, including areas of quantitative health research.

## Triangulation of methods and data

In their article, Gutierrez et al. outline the utility of triangulation in biomedical research, and provide us with the tools to rise to the challenge. Focusing on triangulation of methods, they provide different examples to guide us, from instrument-based methods (e.g., Mendelian randomisation and natural experiments), to bias demonstration and quantification techniques (e.g., negative exposure or outcome controls). The article nicely outlines how these different approaches all come with their own strengths and limitations. Even the ‘holy grail’ of evidence-based medicine, the randomised controlled trial, is not without risk of selection bias due to attrition, and often hampered by poor representativeness [4, 5]. As eloquently summarised by the authors: “The central tenet of evidence triangulation is to identify the most important weaknesses for any given study approach […] and, if necessary, to identify which new sources of evidence that do not share these weaknesses are required”. In other words, we look to change a certain causal assumption, while keeping others constant, to estimate its impact on the observed association. The authors rightfully caution that concordance between methods might still reflect similar bias. This could be characteristic to the method, but also be a trait of the data. For example, misclassification of outcome in one data source might bias various methods in the same direction. Triangulation therefore benefits from diversity in applied methods as well as data.

## Comparable studies and comparable results

Gutierrez et al. demonstrate that the process of triangulation relies on many of the same principles as meta-analysis. Comparability of estimands, definitions of exposure and outcome, and population differences all are important when considering consistency of the presented evidence. With advances in methodology, we start to move beyond qualitative comparisons to translating estimands in—for example—Mendelian randomisation, to those from regression discontinuity or trials. The underlying assumptions are still challenging to meet, and different methods may not necessarily measure the exact same causal effect. Nevertheless, the movements towards quantitative triangulation carries a promise of more direct comparability between studies. It raises the question when results are ‘similar enough’ to be considered consistent. For this, we must assume that the difference between the biases of two estimators is sufficiently large (or the random error sufficiently small) not to confuse differential bias for random error [6]. The comparison of effects across studies therefore needs to account for precision too. As greater random error increases the magnitude of bias needed to conclude that results are divergent, proper triangulation relies on well-powered studies.

## Distinguishing bias from context

In the absence of random error, arguably the biggest challenge in triangulation is to differentiate between issues of internal and external validity. Gutierrez et al. rightly highlight that “[c]omparing estimates across populations with different confounding structures is a valuable evidence triangulation approach to reveal bias, but interpreting such comparisons requires asking whether the true causal effect might differ across the populations.” After all, if we suspect effects to vary between populations, we cannot tell if estimates are discordant because of bias or heterogeneity. Conversely, the importance of the context in which our research takes place can only be discerned if we compare various data sources with similar internal validity. From an epistemic perspective, this leaves us trapped between a rock and a hard place. But we might wiggle our way out. Application of different methods in the same dataset aid to pinpoint the most likely and most prominent sources of bias [7]. In turn, these might guide us to understand whether differences in results across settings are due most likely to bias or context. While researchers may often look for concordance between methods and data, discordance is not a nuisance. Rather, it surfaces the complexity of the research question, invites us to delve deeper into its understanding, and may unveil valuable insights in effect heterogeneity. To fulfil this promise of triangulation requires sufficient diversity in methods as well as data, and continued investment in high-quality data collection worldwide to avoid overreliance on a small number of (publicly available) datasets from high-income countries [8]. Efficient use of electronic health record data may support this, but from the viewpoint of triangulation, routinely-collected healthcare data alone are unlikely to fill the void.

## When and how to triangulate?

Gutierrez et al. provide a valuable toolbox and considerations to support triangulation approaches in our daily academic routine. What remains, is the consideration to either perform methods for triangulation within same the study (i.e., published in the same research article) or across different scientific endeavours. The former could lead to more robust conclusions, avoiding spurious recommendations and potentially waste of resources spent by readers on subsequent studies in dead-end alleys. Yet, a single report of fewer than four thousand words may not do justice to multiple methods and analyses. As touched upon by the authors, an integrated approach may also suffer from confirmation bias on the side of the investigators [9], or selective reporting of concordant results [6], underlined by lower success rates of independent replications compared to those by the original investigators [10]. As such, triangulation of investigators too, may be helpful to draw robust conclusions [2], supporting the involvement of additional, more or less independent researchers in different parts of one’s project. Regardless, the notion of triangulation could turn any article’s discussion section into a much more valuable read, moving beyond statements like “more research is needed” towards a clearer formulation of what type of evidence is needed to strengthen or refute scientific theory.

## Beyond methodology and data

This theoretical framework in which we perceive the world around us, has a large impact on the interpretation of empirical observations. The pioneers in triangulation favoured explicit application of different perspectives, to examine which theory fits best with the empirical data [2]. Such triangulation of theory could prevent paradigms from enduring longer than necessary. Clearly, this is no easy task. Until the seminal work of William Harvey in 1628 [11], the humoral theory prevailed over the circulation of the blood for a long time, despite various incongruent observations. It led William Osler to remark about the esteemed 16th century anatomist Fabricius, “[h]ow […] a teacher of such wide learning and such remarkable powers of observation, could have been so blinded as to overlook the truth which was tumbling out, so to speak, at his feet, is to us incomprehensible. But his eyes were sealed, and […] we read of the hopeless struggle of these great men to escape from slavish submission to authority [of their time]” [12]. A more recent example of triangulation of theory pertains to the characteristic decreases in amyloid-β_42_ in the cerebrospinal fluid of patients with Alzheimer’s disease. These are traditionally interpreted as a mere consequence of accumulation of harmful deposition of amyloid-β_42_ in the brain. Yet, a different interpretation of the same data recently suggested that low amyloid-β_42_ in the cerebrospinal fluid might actually reflect a functional deficit of amyloid-β_42_, potentially prompting an entirely new line of clinical therapy [13]. While the verdict is still out, it exemplifies how our conclusions depend on the lens through which we observe the data. Triangulation of theory can help us to truly treasure the exceptions and discrepancies in our observations, which frequently point us towards new insights.

## Concluding remarks

All in all, the use of multiple methods and data sources, ideally by different investigators, interpreted in the perspective of different theoretical frameworks, describes the essence of empirical science. Better application and integration of triangulation can enhance robustness of the conclusions from scientific research. Whilst this may sound as self-evident to many methodologists, one does not need to look far in biomedical literature to find flawed interpretations of data, careless conclusions, and failures to acknowledge important caveats. The article by Gutierrez et al. can also be considered a call to methodologist to spread this way of thinking into the capillaries of medical academia, as committed missionaries of a triangulation philosophy. Part of their mantra should be, as carefully outlined in the article, that none of the tools by themselves provides a definitive answer, but in their combination lies the strength to come to a closer approximation of the truth, and the context in which it applies.
